# Mechanical performance of a commercial knowledge-based VMAT planning for prostate cancer

**DOI:** 10.1186/s13014-018-1114-y

**Published:** 2018-08-31

**Authors:** Mikoto Tamura, Hajime Monzen, Kenji Matsumoto, Kazuki Kubo, Masakazu Otsuka, Masahiro Inada, Hiroshi Doi, Kazuki Ishikawa, Kiyoshi Nakamatsu, Iori Sumida, Hirokazu Mizuno, Do-Kun Yoon, Yasumasa Nishimura

**Affiliations:** 1 Department of Medical Physics, Graduate School of Medical Science, Kindai University, 377-2, Ohno-Higashi, Osakasayama, Osaka 589-8511 Japan; 2 Department of Radiation Oncology, Faculty of Medicine, Kindai University, 377-2, Ohno-Higashi, Osakasayama, Osaka 589-8511 Japan; 3 Department of Radiation Oncology, Graduate School of Medicine, Osaka University, 2-2, Yamada-oka, Suita, Osaka 565-0071 Japan; 4 Department of Biomedical Engineering and Research Institute of Biomedical Engineering, College of Medicine, Catholic University of Korea, 505, Banpo-dong, Seocho-gu, Seoul, 137-701 Korea

**Keywords:** Knowledge-based treatment planning, Prostate cancer, Mechanical performance, Dosimetric accuracy

## Abstract

**Background:**

This study clarified the mechanical performance of volumetric modulated arc therapy (VMAT) plans for prostate cancer generated with a commercial knowledge-based treatment planning (KBP) and whether KBP system could be applied clinically without any major problems with mechanical performance.

**Methods:**

Thirty consecutive prostate cancer patients who underwent VMAT using extant clinical plans were evaluated. The mechanical performance and dosimetric accuracy of the single optimized KBPs, which were trained with other 51 clinical plans, were compared with the clinical plans. The mechanical performance metrics were mean field area (MFA), mean asymmetry distance (MAD), cross-axis score (CAS), closed leaf score (CLS), small aperture score (SAS), leaf travel (LT), modulation complexity score (MCS_v_), and monitor unit (MU). The γ passing rates were evaluated with ArcCheck and EBT3 film.

**Results:**

The mean mechanical performance metrics (clinical plan vs. KBP) were as follows: 18.28 cm^2^ vs. 17.25 cm^2^ (MFA), 21.08 mm vs. 20.47 mm (MAD), 0.54 vs. 0.55 (CAS), 0.040 vs. 0.051 (CLS), 0.20 vs. 0.23 (SAS_5mm_), 458.5 mm vs. 418.8 mm (LT), 0.27 vs. 0.27 (MCS_v_), and 618.2 vs. 622.1 (MU), respectively. Significant differences were observed for CLS and LT. The average γ passing rates (clinical plan vs. KBP) were as follows: 99.0% vs. 99.1% (3%/3 mm) and 92.4% vs. 92.5% (2%/2 mm) with ArcCHeck, and 99.5% vs. 99.4% (3%/3 mm) and 95.2% vs. 95.4% (2%/2 mm) with EBT3 film, respectively.

**Conclusions:**

The KBP used lower multileaf collimator (MLC) travel and more closed or small MLC apertures than the clinical plan. The KBP system of VMAT for the prostate cancer was acceptable for clinical use without any major problems.

## Background

In intensity-modulated radiotherapy (IMRT) and volumetric modulated arc therapy (VMAT), variations in knowledge and experience can lead to significant differences in the plan quality and may compromise the gains of high-precision radiotherapy [1, 2]. Knowledge-based treatment planning (KBP) is an approach to reduce the differences in plan quality and improve planning consistency [1]. A commercial KBP module, RapidPlan (Varian Medical Systems, Palo Alto CA, USA) has been released for the Eclipse (Varian) treatment planning system (TPS). KBP uses a statistical model generated from a library of clinically accepted plans with consistent high quality to train dose volume histograms (DVHs) [1, 3]. This model predicts an achievable DVH range and generates dose volume objectives for IMRT and VMAT plan optimization [1, 3].

Many studies have reported the KBP can generate the better or comparable dosimetric results in some anatomical sites [1–9]. Hussein et al. described the KBP was able to produce IMRT and VMAT treatment plans for prostate cancer with a single optimization, with sparing and conformity better than or comparable to a clinical plan [1]. In addition, Ueda et al. suggested the KBP could also reproduce the dose distributions based on the experience of institutions in the case of sharing the KBP model, although it was necessary to verify the registered DVH curves could match the plan design of each institution [10]. However, Kubo et al. found that KBP may encounter difficulties with high numbers of MUs and high modulation complexity [11]. At this time, we focused on the mechanical performance of the KBP, which has not been delineated. Additionally, dosimetric accuracy can be affected by mechanical performance [12, 13]. VMAT delivery requires extremely precise mechanical performance for a linear accelerator (Linac) due to the simultaneous gantry speed, dose rate, and multileaf collimator (MLC) aperture shape variations; this makes patient-specific quality assurance (QA) an essential prerequisite for actual patient treatment [13, 14]. The purpose of this study was to clarify the mechanical performance of the VMAT plans generated by the KBP for prostate cancer and verify patient-specific QA to assess KBP’s appropriateness in the clinical setting.

## Methods

### Clinical plan and KBP

The cases of 30 consecutive prostate cancer patients (between September 2016 and March 2017) who underwent VMAT with clinical plans were analyzed. We trained the KBP system with the other consecutive 51 cases of T1–T2c prostate cancer treated between August 2015 and August 2016. Written informed consent was obtained from all patients and the Institutional Ethics Committee approved this study (institutional review board number: 29–133). The KBP model configuration and training process is well-explained in the literature [5, 7, 11]. The clinical target volume (CTV) was defined as the prostate and seminal vesicle and delineated by experienced radiation oncologists. The planning target volume (PTV) was defined with a 6 mm posterior margin and a 10 mm margin in all other directions added to the CTV to reduce the dose at the prostate-rectal interface. The organ at risks (OARs) were rectal wall and bladder wall. The rectum was delineated up to 1.0 cm above and below the PTV. The rectal wall and bladder wall were delineated 4.0 mm inside the outer surface of rectum and bladder, respectively. The prescribed dose was 78 Gy in 39 fractions to 95% of the volume of the PTV minus rectum (PTV − R) using 10 MV photon beams and 2 full arcs VMAT (Gantry angle: 181°–179°, clockwise and 179°–181°, counterclockwise, collimator angles: 30° and 330°). The control point spacing was 2° angular separation. All VMAT plans were calculated with the Varian Analytic Anisotropic Algorithm (AAA) [15] using the Eclipse treatment planning system ver. 13.6 for a TrueBeam (Varian) with a Millennium 120 MLC. The goals of the treatment plan in our institution were as follows: The maximum dose (D_max_) was < 110% and the mean dose (D_mean_) was 99–103% of the prescribed dose for the PTV − R; V_40 Gy_ <  60%, V_60 Gy_ <  35%, V_70 Gy_ <  25%, and V_78 Gy_ <  1% for the rectal wall; V_40 Gy_ <  60% and V_70 Gy_ <  35% for the bladder wall [10, 16]. The overlap region between PTV and rectal wall was covered with the 90% isodose line.

The geometry and dosimetry of the PTV − R, rectum, and bladder were registered in the KBP library. In this model, the geometry or dosimetry outliers were not excluded since the removal of statistical outliers had no significant impact on establishing the model [1]. Then, we performed the dose calculation using KBP with the single optimization in the 30 consecutive clinical cases.

### KBP validation

The following parameters were compared between the 30 clinical plans and the 30 KBPs:

1. The maximum (D_max_), minimum (D_min_), and mean dose (D_mean_) of the PTV–R volume
2. Homogeneity index (HI; defined as 100 *×* [*D*_*2%*_ − *D*_*98%*_]/*D*_*50%*_), where *D*_*98%*_, *D*_*2%*_, and *D*_*50%*_ are doses received by 98%, 2%, and 50% of the PTV − R, respectively [17]
3. The 95% isodose conformity index (CI_95_; defined as *V*_*95%*_/*V*_*PTV − R*_), where *V*_*95%*_ is volume covered by 95% of the prescribed dose (74.1 Gy) and *V*_*PTV − R*_ is PTV–R volume [1]
4. Dose-volume parameters of the rectal wall: V_40 Gy_, V_60 Gy_, V_70 Gy_, and V_78 Gy_
5. Dose-volume parameters of the bladder wall: V_40 Gy_ and V_70 Gy_

### Mechanical performance metrics

For each of the 30 clinical plans and the 30 KBPs, 8 mechanical performance metrics were verified as shown in Table 1: 1. mean field area (MFA), 2. mean asymmetry distance (MAD), 3. cross-axis score (CAS), 4. closed leaf score (CLS), 5. small aperture score (SAS) [18–20], 6. leaf travel (LT), 7. modulation complexity score for VMAT (MCS_v_), and 8. total MUs [12, 13].

**Table 1:** Mechanical performance metrics

Mechanical performance metrics		Abbreviation	Description
1	Mean field area	MFA	Mean of the field area weighted according to the MU at each control point
2	Mean asymmetry distance	MAD	Mean lateral distance between open MLC leaf pairs apertures and the central axis
3	Cross-axis score	CAS	Proportion of MLC leaf pairs crossing the central axis within the jaw aperture
4	Closed leaf score	CLS	Proportion of MLC leaf pairs entirely closed within the jaw aperture
5	Small aperture score	SAS	Proportion of open MLC leaf pairs separated by less than the given thresholds (2, 5, 10, and 20 mm in this study)
6	Leaf travel	LT	Averaged over all in-field moving leaves
7	Modulation complexity score for VMAT	MCS_v_	Sum over all control points of the product of the aperture area variability (AAV), leaf sequence variability (LSV), and normalized MU
8	Monitor unit	MU	Sum of monitor unit value for a plan

### Patient-specific QA

The gamma index evaluation for patient-specific QA has been a standard technique used to evaluate measured distributions in commercial detector systems and gafchromic film against the dose distributions predicted by TPSs [21]. We employed ArcCheck (SunNuclear, Melbourne FL, USA) as a commercial detector system for patient-specific QA. The film dosimetry was also performed. The EBT3 gafchromic film (Ashland ISP Advanced Materials, Bridgewater, NJ, USA) was inserted at the sagittal plane in center of the ArcCheck. Irradiated films were scanned using Epson ES-10000G scanner (Epson Corp., Nagano, Japan) at least 24 h after irradiation with a resolution of 150 dpi from red channel. A calibration curve, film optical density versus dose, was determined for a range from 0 to 300 cGy. For analysis, the DoseLab ver. 6.70 (Mobius Medical Systems, TX, USA) was employed. An ionization chamber (CC01, IBA, Schwarzenbruck, Germany) was used for measurement of absorbed dose at the isocenter in the ArcCheck (source-to-axis distance (SAD) = 100 cm). Then we evaluated the differences in dose distribution and point dose at isocenter among plans and measurements for each clinical plan and KBP. The difference of dose distribution was evaluated using the passing rate of γ index with two tolerances in terms of dose difference (DD) and distance to agreement (DTA) (3%/3 mm and 2%/2 mm) with a threshold at 10%.

### Statistical analysis

The data are expressed as the median and interquartile ranges (first quartile, third quartile), unless otherwise indicated. The Wilcoxon signed rank test was used to compare continuous variables and trends between the KBPs and the clinical plans. All statistical analyses were performed using R ver. 3.4.2 (The R Foundation for Statistical Computing, Vienna, Austria) and *p* <  0.05 was considered statistically significant. Peason’s correlation was considered weak for *r* <  0.4, moderate for 0.4 ≤ *r* ≤ 0.7, and strong for *r* > 0.7.

## Results

### KBP validation

**Fig. 1:**
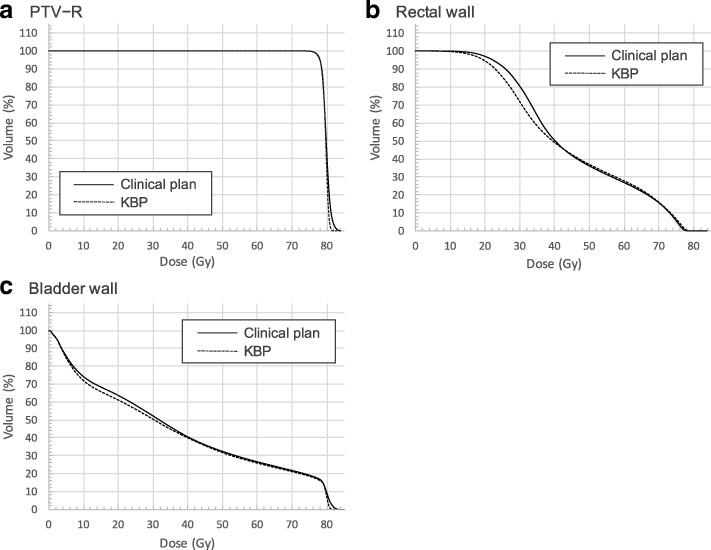
The average DVHs of the clinical plan and the KBP for PTV − R (**a**), rectal wall (**b**), and bladder wall (**c**). For the PTV − R of the KBP, the D_max_ was lower than those of the clinical plans, which improved the homogeneity. The V_78 Gy_ for rectal wall of the KBP was higher than those of the clinical plans, however it was < 1% for all cases

Table 2 summarizes the results of comparisons between the clinical plan and the KBP of D_max_, D_min_, D_mean_, HI and CI_95_ for PTV − R, dose-volume parameters for rectal wall and bladder wall. For the PTV − R, the D_min_ and the D_mean_ were comparable between the clinical plan and the KBP, and the D_max_ of the KBP was significantly lower than those of the clinical plan (*p* <  0.01). The homogeneity of PTV − R of the KBP was significantly better than those of the clinical plan (*p* = 0.03). In addition, the PTV − R coverage of the KBP was more conformal as indicated by the CI_95_ being significantly lower than the CI_95_ of the clinical plan (*p* <  0.01). For the rectal wall and the bladder wall, the dose-volume parameters of the KBP were comparable to those of the clinical plan except for the V_78 Gy_ for the rectal wall. The V_78 Gy_ for the rectal wall of the KBP was higher than those of the clinical plan; however, it was < 1% for all cases. The mean DVHs for the clinical plan and the KBP were shown in Fig. 1. The KBP was able to generate better or comparable dosimetric results compared to clinical plan using our training model.

**Table 2:** The summary of dose-volume parameters in the clinical plan and KBP

	Parameter	Clinical goal	Clinical plan	KBP	*p*-value
PTV − R	D_max_ (%)	< 110%	106.40 (105.83, 107.68)	105.60 (105.30, 106.10)	< 0.01
	D_min_ (%)	–	91.55 (89.88, 93.98)	91.75 (89.63, 92.90)	0.33
	D_mean_ (%)	99–103%	102.20 (102.00, 102.68)	102.05 (101.93, 102.20)	0.07
	HI	–	5.08 (4.54, 6.17)	4.76 (4.52, 4.89)	0.03
	CI_95_	–	1.27 (1.23, 1.32)	1.18 (1.17, 1.20)	< 0.01
Rectal wall	V_40 Gy_ (%)	< 60%	49.10 (45.38, 55.20)	49.60 (46.03, 52.93)	0.94
	V_60 Gy_ (%)	< 35%	26.05 (22.80, 29.20)	27.60 (24.08, 30.95)	0.20
	V_70 Gy_ (%)	< 25%	15.00 (12.40, 16.88)	16.40 (14.70, 16.90)	0.13
	V_78 Gy_ (%)	< 1%	0.0045 (0.00, 0.040)	0.28 (0.18, 0.47)	< 0.01
Bladder wall	V_40 Gy_ (%)	< 60%	40.65 (34.93, 47.85)	38.95 (27.90, 52.60)	0.82
	V_70 Gy_ (%)	< 35%	22.30 (17.15, 27.25)	21.00 (15.95, 27.43)	0.67

Abbreviations: *PTV − R* volume of the planning target volume minus rectum, *HI* homogeneity index, *CI*_*95*_ 95% isodose conformity index

### Mechanical performance

**Fig. 2:**
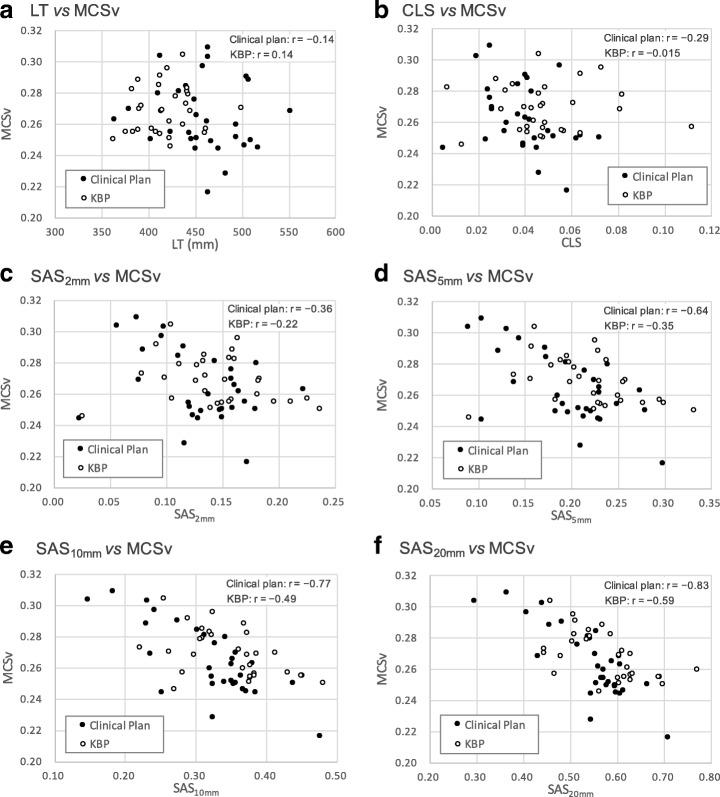
The relationships between MCS_v_ and LT (**a**), CLS (**b**), SAS_2mm_ (**c**), SAS_5mm_ (**d**), SAS_10mm_ (**e**), and SAS_20mm_ (**f**) (closed circle: clinical plan, open circle: KBP). Abbreviations: *MCS*_*v*_ modulation complexity score for VMAT, *LT* leaf travel, *CLS* closed leaf score, *SAS* small aperture score

Table 3 summarizes the mechanical performance metrics of MFA, MAD, CAS, CLS, SAS (thresholds: 2, 5, 10, and 20 mm), LT, MCS_v_, and MU for the clinical plan and the KBP. The MFA, MAD, CAS, MCS_v_ and MU of the KBP were almost equal to those of the clinical plan. On the other hand, there were significant differences for CLS (*p* = 0.03) and LT (*p* <  0.01). The KBP used more closed leaves and less MLC travel compared to the clinical plan. The SASs for each threshold of the KBP tended to be higher than those for the clinical plans, although there were no significant differences. This suggests that KBP system may use more small apertures in the MLCs than the clinical plan at each control point. In addition, the relationships between MCS_v_ and LT, CLS, and SAS were shown in Fig. 2. The LT, CLS, and SAS_2mm_ were not correlated to the MCS_v_ in Fig. 2a–c. On the other hand, negative correlations were observed between MCS_v_ and SASs for threshold ≥10 mm in Fig. 2e, f.

**Table 3:** The results of mechanical performance metrics for the clinical plan and KBP

	Clinical Plan				KBP				*p*-value
	Mean	Max	Min	SD	Mean	Max	Min	SD	
MFA (cm^2^)	18.28	25.07	12.39	3.34	17.25	22.89	12.15	2.80	0.23
MAD (mm)	21.08	25.04	17.27	2.14	20.47	24.49	16.88	1.92	0.38
CAS	0.54	0.63	0.34	0.07	0.55	0.66	0.41	0.07	0.46
CLS	0.040	0.072	0.0050	0.015	0.051	0.16	0.0070	0.027	0.03
SAS_2mm_	0.13	0.22	0.022	0.042	0.15	0.24	0.026	0.044	0.20
SAS_5mm_	0.20	0.30	0.089	0.053	0.23	0.33	0.091	0.087	0.05
SAS_10mm_	0.32	0.48	0.15	0.071	0.35	0.48	0.22	0.063	0.07
SAS_20mm_	0.54	0.71	0.29	0.087	0.58	0.77	0.44	0.081	0.11
LT (mm)	458.51	550.80	362.80	41.62	418.81	499.60	362.10	29.39	< 0.01
MCS_v_	0.27	0.31	0.22	0.023	0.27	0.30	0.25	0.015	0.24
MU	618.24	738.80	475.70	64.77	622.12	672.60	572.40	25.58	0.76

Abbreviations: *MFA* mean field area, *MAD* mean asymmetry distance, *CAS* cross-axis score, *CLS* closed leaf score, *SAS* small aperture score, *LT* leaf travel, *MCS*_*v*_ modulation complexity score for VMAT, *MU* monitor unit

### Patient-specific QA

Table 4 shows the γ passing rate of each criterion for the clinical plan and the KBP. No significant differences were observed between the clinical plan and the KBP in the γ passing rate with ArcCheck and EBT3 film. The γ passing rates of the clinical plan and the KBP were > 90% for all cases for commonly used criteria of 3%/3 mm [22–24]. For more stringent 2%/2 mm, the cases of the clinical plan and the KBP in which the γ passing rates were > 90% with the ArcCheck were 24 and 25 cases in 30 cases, respectively. On the other hand, the cases of both the clinical plan and the KBP in which the γ passing rates were > 90% with EBT3 film were 28 cases in 30 cases. No mechanical performance metrics were correlated to the γ passing rate. The differences in absorbed dose at the isocenter for measurement compared to the TPS were − 0.40% (− 0.86%, 0.023%) and − 0.29% (− 0.50%, − 0.083%) for the clinical plan and the KBP, respectively (*p* = 0.48).

**Table 4:** The results of the γ passing rate for the clinical plan and KBP with ArcCheck and EBT3 film

	Clinical Plan				KBP				*p*-value
	Mean	Max	Min	SD	Mean	Max	Min	SD	
ArcCheck									
2%/2 mm	92.4%	97.5%	87.8%	2.3	92.5%	96.2%	87.8%	2.4	0.69
3%/3 mm	99.0%	99.8%	97.5%	0.6	99.1%	100.0%	97.4%	0.7	0.46
EBT3 film									
2%/2 mm	95.2%	99.0%	88.7%	2.6	95.4%	99.1%	87.3%	3.1	0.65
3%/3 mm	99.5%	100.0%	97.8%	0.5	99.4%	100.0%	97.4%	0.7	0.85

## Discussion

In this study, we clarified the mechanical performance and evaluated the dosimetric accuracy of the VMAT generated by the KBP for prostate cancer. The KBP system can be used in clinical practice without any major problems, although the KBP created dose distributions by use of lower MLC travel and more closed or small MLC apertures compared to the clinical plan.

In the KBP model validation, Table 2 and Fig. 1 show the dose volume parameters of the KBP with the single optimization for PTV − R were better or comparable, and for rectal wall and bladder wall were comparable, to the clinical plan, respectively. The KBP also had higher homogeneity and conformity for PTV − R compared to the clinical plan. However, this model could contain a limited number of structures (e.g. femoral head, sacrum, anal canal, and bulbus penis).

In mechanical performance, the CLS and the LT values of the KBP were significantly different from the clinical plan as shown in Table 3. Additionally, the SASs for all thresholds of the KBP were higher than those of the clinical plan, although not to a significant degree. The KBP system might use lower MLC travel, and more closed or small MLC apertures at each control point than the clinical plan. The negative correlations between MCS_v_ and SASs for threshold ≥10 mm were shown in Fig. 2e–f. The modulation complexity was increased with the proportion of small apertures increasing, which was also described by Crowe et al. [20]. Therefore, one full arc VMAT plan generated by the KBP might increase the MU and the modulation complexity compared to the clinical plan [11]. On the other hand, two full arcs VMAT plans generated by the KBP might decrease the MU and the modulation complexity. Hussein et al. also showed the MU and modulation complexity were not difference between the KBP and clinical plan for two full arcs VMAT plans [1].

The γ passing rates for all criteria of DD and DTA were not significantly different between the clinical plan and the KBP, and all cases of the clinical plan and the KBP had the γ passing rates of more than 90% for AAPM recommending criteria of 3%/3 mm [23] as shown in Table 4, although the mechanical performance of the KBP did not correspond to that of the clinical plan. A patient-specific QA passed if the γ passing rate was > 90% of points achieving the 3%/3 mm criterion, which is acceptable in the clinical setting [22–24], although some reports describe more stringent criteria such as 2%/2 mm [25–27]. It is necessary to understand the sensitivity and limitations of the gamma index analysis combined with the equipment in use [21].

## Conclusions

The KBP with the single optimization used lower MLC travel and more closed or small MLC apertures compared to the clinical plan. The VMAT plan generated by the KBP for prostate cancer could be applied clinically without any major problems.

## Abbreviations

AAA: Analytic Anisotropic Algorithm
AAV: Aperture area variability
CAS: Cross-axis score
CI_95_: 95% isodose conformity index
CLS: Closed leaf score
CTV: Clinical target volume
DD: Dose difference
DTA: Distance to agreement
DVH: Dose volume histogram
HI: Homogeneity index
IMRT: Intensity-modulated radiotherapy
KBP: Knowledge-based treatment planning
Linac: Linear accelerator
LSV: Leaf sequence variability
LT: Leaf travel
MAD: Mean asymmetry distance
MCSv: Modulation complexity score for VMAT
MFA: Mean field area
MLC: Multileaf collimator
MU: Monitor unit
OAR: Organ at risk
PTV − R: Volume of the PTV minus rectum
PTV: Planning target volume
QA: Quality assurance
SAD: Source-to-axis distance
SAS: Small aperture score
TPS: Treatment planning system
VMAT: Volumetric modulated arc therapy
